# Economic Burden Associated With Extended-Release vs Immediate-Release Drug Formulations Among Medicare Part D and Medicaid Beneficiaries

**DOI:** 10.1001/jamanetworkopen.2020.0181

**Published:** 2020-02-28

**Authors:** Andrew Sumarsono, Nathan Sumarsono, Sandeep R. Das, Muthiah Vaduganathan, Deepak Agrawal, Ambarish Pandey

**Affiliations:** 1 Department of Internal Medicine, The University of Texas Southwestern Medical Center, Dallas; 2 Department of Pediatrics, Stanford University, Palo Alto, California; 3 Division of Cardiology, Department of Internal Medicine, The University of Texas Southwestern Medical Center, Dallas; 4 Division of Cardiology, Department of Medicine, Brigham and Women’s Hospital, Boston, Massachusetts; 5 Division of Gastroenterology and Hepatology, Department of Internal Medicine, University of Texas at Austin, Dell Medical School, Austin

## Abstract

**Importance:**

The United States spends more money on medications than any other country. Most extended-release drugs have not consistently shown therapeutic or adherence superiority, and switching these medications to less expensive, generic, immediate-release formulations may offer an opportunity to reduce health care spending.

**Objective:**

To evaluate Medicare Part D and Medicaid spending on extended-release drug formulations and the potential savings associated with switching to generic immediate-release formulations.

**Design, Setting, and Participants:**

This cross-sectional study used the 2012 to 2017 Medicare Part D Drug Event and Medicaid Spending and Utilization data sets to analyze 20 extended-release drugs with 37 Medicare formulations and 36 Medicaid formulations. Only cardiovascular, diabetes, neurologic, and psychiatric extended-release drugs saving at most 1 additional daily dose compared with their immediate-release counterparts were included. Extended-release drugs with therapeutic superiority were excluded. Analyses were conducted from January to December 2019.

**Main Outcomes and Measures:**

Estimated Medicare Part D and Medicaid savings from switching extended-release to immediate-release drug formulations between 2012 and 2017.

**Results:**

Of the 6252 drugs screened for eligibility from the 2017 Medicaid Drug Utilization database and the 2017 Medicare Part D database, 67 drugs with extended-release formulations that were identified in the Medicare data set (20 distinct drugs with 37 formulations [19 brand, 18 generic]) were included in the analysis. In 2017, Medicare Part D spent $2.2 billion and Medicaid spent $952 million (a combined $3.1 billion) on 20 extended-release drugs. Between 2012 and 2017, Medicare Part D and Medicaid spent $12 billion and $5.9 billion, respectively, on extended-release formulations. Switching from brand-name to generic extended-release formulations was estimated to be associated with a $247 million reduction in Medicare spending and $299 million reduction in Medicaid spending in 2017, whereas switching all brand-name and generic extended-release formulations to immediate-release formulations in both Medicare and Medicaid was estimated to reduce spending by $2.6 billion ($1.8 billion for Medicare and $836 million for Medicaid) in 2017. During the study period, the estimated spending reduction associated with switching all patients receiving extended-release formulations (brand name extended-release and generic extended-release) to generic immediate-release formulations was $13.7 billion ($8.5 billion from Medicare and $5.2 billion from Medicaid).

**Conclusions and Relevance:**

The findings suggest that switching from extended-release drug formulations to therapeutically equivalent immediate-release formulations when available represents a potential option to reduce Medicare and Medicaid spending.

## Key Points

**Question:**

How much do Medicare and Medicaid spend on extended-release drug formulations, and what would be the potential savings associated with switching to immediate-release formulations?

**Findings:**

In this cross-sectional study of 20 extended-release drugs, Medicare Part D and Medicaid spent a combined $3.1 billion in 2017. Switching to immediate-release drug formulations was associated with an estimated $2.6 billion reduction in spending in 2017 and a $13.7 billion reduction from 2012 to 2017.

**Meaning:**

The findings suggest that substitution of therapeutically equivalent extended-release drug formulations with immediate-release formulations represents a possible option to reduce Medicare and Medicaid spending.

## Teaser

This cross-sectional study assesses Medicare Part D and Medicaid spending on extended-release drug formulations and the potential savings associated with switching to generic immediate-release drug formulations.

## Introduction

Medication costs compose up to 20% of US health care expenditures and are up to 3-fold higher per capita than in other high-income countries.^1^ Previous studies have evaluated strategies such as switching brand-name drugs to their generic counterparts in order to lower costs.^2^ However, the economic consequences of switching from extended-release (ER) drug formulations to generic (therapeutically comparable) immediate-release (IR) drug formulations have not been studied, to our knowledge. Although ER drug formulations have been traditionally used to lower pill burden and, in theory, to achieve greater adherence to therapies, data on their overall effectiveness in improving adherence compared with IR formulations are mixed and vary by drug type. Examples of this include equal adherence between carvedilol ER and carvedilol IR formulations but improved adherence with methylphenidate ER vs methylphenidate IR formulations.^3,4^ A Cochrane review^5^ that evaluated drug administration schedule and medication adherence found inadequate evidence to recommend a less frequent administration schedule but noted the possibility of improved adherence if the regimen was reduced by 2 or more daily doses. The prospect of significantly increasing the number of daily administrations to reduce cost may be impractical for many patients. Accordingly, we quantified the cost of ER formulations saving at most 1 additional daily dose compared with their IR counterparts and estimated the cost savings from switching to therapeutically comparable generic IR formulations among Medicare and Medicaid beneficiaries.

## Methods

For this cross-sectional study, the primary analysis was performed using the publicly available 2012-2017 Medicare Part D Prescription Drug Event data set,^6^ which contains medication expenditures for approximately 70% of all Medicare beneficiaries, and the Medicaid Spending and Utilization Data set,^7^ which contains national-level drug utilization data for outpatient drugs paid for by state Medicaid agencies. Because all data were deidentified and publicly available, The University of Texas Southwestern Human Research Protection Program determined this project did not meet criteria for human subjects’ research and did not require institutional review board approval. This study followed the Strengthening the Reporting of Observational Studies in Epidemiology (STROBE) reporting guideline.

**Figure 1.**
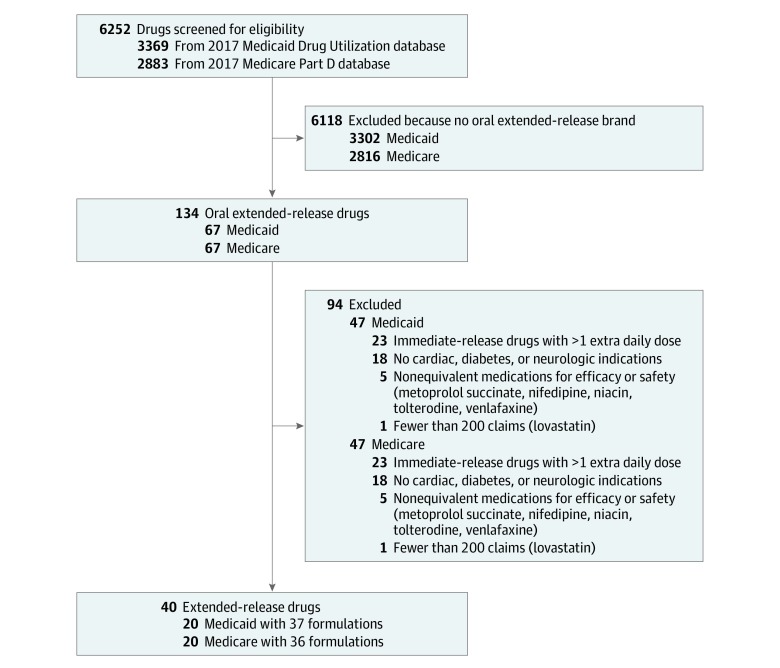
Flow Diagram of Medicare and Medicaid Drug Selection Medicaid-only formulations: Dexedrine (dextroamphetamine sulfate), Fortamet (metformin hydrochloride); Medicare-only formulations: Lescol XL (fluvastatin), Rhythmol SR (propafenone hydrochloride), Zenzedi (dextroamphetamine sulfate).

The Medicare data set contains 2883 formulations, whereas the Medicaid data set contains 3369 formulations (Figure 1). Drugs with ER formulations in the data sets were identified independently by 2 of us (A.S and N.S.). The ER formulations with disease-specific indications or well-established therapeutic or adverse effect superiority compared with generic IR formulations were excluded (niacin, nifedipine, metoprolol succinate, venlafaxine hydrochloride, and tolterodine). Only drugs with cardiovascular, diabetes, neurologic, and psychiatric indications were included. The study included only oral medications with ER and generic IR formulations with more than 200 claims in 2017 (Figure 1). Only drugs with ER formulations saving at most 1 additional daily dose compared with their generic IR counterparts were included. We extracted data on drug name, number of claims, number of units dispensed, and total expenditure incurred (overall and per claim) for the ER and generic IR formulations. The Medicare data set–reported spending included both beneficiary and government contributions, whereas the Medicaid data set reported both federal and state reimbursement to pharmacies. These data include only total costs to both Medicare and Medicaid and do not account for out-of-pocket costs for the patient. To conservatively estimate the cost, we assumed that all patients who switched to generic IR formulations would use the maximum number of daily administrations recommended. We calculated the savings with the following formula: estimated savings = (daily ER drug dose per unit price − daily IR drug dose per unit price) × total ER drug units dispensed.

Temporal trends in annual expenditures on all drugs with available ER formulations and the estimated expenditure substituting generic IR formulations were also assessed between 2012 and 2017. Because 100% interchange between ER and generic IR formulations may not be possible, we performed a sensitivity analysis to estimate overall cost reduction if 25%, 50%, or 75% of all ER formulations were substituted with generic IR formulations. We additionally performed a second sensitivity analysis to evaluate whether using a per-claim–level analysis would yield different results than a per-unit–level analysis.

We estimated the Medicare postrebate spending using the mean 26.3% for all brand-name cardiovascular drug classes and the mean 13.0% for all brand-name central nervous system drug classes noted from the 2014 Medicare Part D Rebate Summary.^8^ Similarly, using the Medicaid Drug Rebate Program, we estimated a 23.1% per-unit rebate for all brand-name formulations and 13.0% per-unit rebate for all generic formulations.^9^

### Statistical Analysis

All dollar amounts are described in 2017 US dollars and have been adjusted for inflation. All analyses were performed in Microsoft Excel, version 16.0 (Microsoft) and Graphpad Prism 7.0 software (Graphpad).

## Results

### Medicare

Of the 6252 drugs screened for eligibility from the 2017 Medicaid Drug Utilization database and the 2017 Medicare Part D database, 67 with ER formulations that were identified in the Medicare data set (20 distinct drugs with 37 formulations [19 brand, 18 generic]) were included in the analysis (Table 1). In 2017, Medicare spent $2.2 billion ($1.9 billion after correction for brand-name drug rebates) on ER formulations ($1347 million on brand-name and $806 million on generic formulations) for 1.5 billion pills at a mean (SD) cost of $1.47 ($2.21) per pill ($13.75 [$7.21] per pill for brand name and $0.59 [$1.23] per pill for generic formulations). Namenda XR (memantine hydrochloride) had highest overall spending ($891 million for 2.5 million claims and 69 million pills), and brand-name ER metformin hydrochloride (Glumetza) had the highest per claim spending ($7619.35 per claim or $74.83 per pill) for Medicare in 2017. The Medicare expenditure for generic IR formulations was $1.2 billion at a mean cost of $14.34 ($15.82) per claim and $0.16 ($0.21) per pill in 2017.

**Table 1.** Per-unit Spending and Potential Savings Associated With Switching ER Drug Formulations to Generic IR Drug Formulations in Medicare Part D, 2017

Medication Type, ER Drug Name	ER Formulation				IR Formulation			Estimated Savings, $US in Millions
	Daily Dose Frequency, Pills/d	Pills Dispensed, No. in Thousands	Mean Spending per Pill, $	Spending, $US in Millions	Generic Name	Daily Dose Frequency, Pills/d	Mean Spending per Pill, $	
**Cardiometabolic**								
Coreg CR	1	7291	8.82	64.3	Carvedilol phosphate	2	0.06	63.2
Fluvastatin ER	1	1320	5.00	6.6	Fluvastatin sodium	1-2	3.00	−1.3
Lescol XL	1	275	9.70	2.7				1.0
Glipizide ER	1	122 616	0.27	33.8	Glipizide	1-2	0.07	16.6
Glipizide XL	1	71 750	0.28	20.1				10.0
Glucotrol XL	1	547	1.99	1.1				1.0
Isosorbide mononitrate ER	1	312 855	0.32	101.6	Isosorbide mononitrate	1-2	0.23	−42.1
Glucophage XR	1	1735	1.04	1.8	Metformin HCl	1-2	0.06	1.6
Glumetza	1	1049	74.83	73.8				78.4
Metformin HCl ER	1	757 381	0.60	320.1				356.2
Actoplus Met XR	1	228	12.77	2.7	Pioglitazone HCl plus metformin HCl	1-2	2.05	2.0
Propafenone HCl ER	2	8162	5.40	44.3	Propafenone HCl	3	0.41	37.4
Rhythmol SR	2	320	12.78	4.1				3.8
**Central Nervous System**								
Dexmethylphenidate HCl ER	1	657	5.25	3.5	Dexmethylphenidate HCl	2	0.76	2.4
Focalin XR	1	101	11.63	1.2				1.0
Zenzedi	1-2	70	6.19	0.4	Dextroamphetamine sulfate	1-3	1.40	0.2
Dextroamphetamine sulfate ER	1-2	3939	2.82	11.1				0.1
Fluvoxamine maleate ER	1	1084	7.22	7.8	Fluvoxamine maleate	2	0.51	6.7
Galantamine ER	1	6063	2.86	17.3	Galantamine HBr	2	1.60	−2.1
Lamictal XR	1	1707	24.21	40.3	Lamotrigine	2	0.17	40.8
Lamotrigine ER	1	5897	8.11	46.8				45.9
Keppra XR	1	3156	8.07	24.9	Levetiracetam	2	0.27	23.8
Levetiracetam ER	1	14 710	0.62	9.0				1.2
Lithobid	2	234	9.39	2.2	Lithium carbonate	3	0.11	2.1
Lithium carbonate ER	2	31 364	0.27	8.3				1.6
Namenda XR	1	67 829	13.14	891.2	Memantine HCl	1-2	0.94	763.4
Oxtellar XR	1	1043	11.29	11.7	Oxcarbazepine	2	0.43	10.9
Paroxetine CR	1	1550	3.67	5.7	Paroxetine HCl	1	0.23	5.0
Paroxetine ER	1	4557	3.71	16.8				14.7
Paxil CR	1	472	6.05	2.9				2.6
Quetiapine fumarate ER	1	11 525	11.02	127.3	Quetiapine fumarate	2	0.37	118.5
Seroquel XR	1	10 039	18.58	188.2				179.2
Qudexy XR	1	112	17.41	1.9	Topiramate	1-2	0.16	1.9
Topiramate ER	1	333	11.84	3.9				3.8
Trokendi XR	1	1158	20.36	23.6				23.2
Ambien CR	1	604	14.28	8.6	Zolpidem tartrate	1	0.27	8.3
Zolpidem tartrate ER	1	12 576	1.75	21.9				15.3

Abbreviations: CR, controlled release; ER, extended release; HBr, hydrobromide; HCl, hydrochloride; IR, immediate release; XR, extended release.

**Figure 2.**
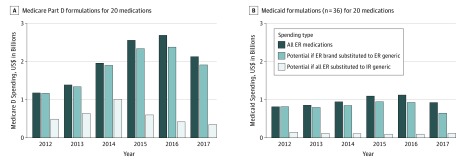
Medicare Part D and Medicaid Spending and Potential Savings Associated With Switching to Less Costly Formulations ER indicates extended release; IR, immediate release.

In 2017, the estimated spending reduction for switching from brand-name ER to generic ER formulations was $247 million ($183 million with rebates). In contrast, switching all (brand and generic) ER formulations to generic IR formulations in 2017 was associated with a $1.8 billion reduction ($1.6 billion after accounting for brand-name drug rebates). Between 2012 and 2017, Medicare Part D spent $12 billion on ER formulations, and switching all patients receiving ER formulations to generic IR formulations was associated with an $8.5 billion reduction in spending ($7.2 billion after correction for brand-name drug rebates) (Figure 2A).

### Medicaid

The same 20 drugs with 36 formulations were included in the Medicaid analysis (Table 2). In 2017, Medicaid spent $952 million on ER formulations ($598 million on brand-name formulations and $354 million on generic formulations) for 294 million pills at a mean cost of $3.24 ($3.88) per pill ($11.16 [$6.94] per pill for brand formulations and $1.47 [$2.61] for generic formulations). Focalin XR (dexmethylphenidate hydrochloride) had the highest total expenditure ($307 million for 865 918 claims of 27 million pills), and Glumetza (metformin) had the highest per-claim spending ($4840.33 per claim and $78.71 per pill) for Medicaid. Medicaid spent $429 million for 35 million claims of generic IR formulations and 2.3 million pills at a mean cost of $12.14 ($10.86) per claim and $0.18 ($0.14) per pill in 2017.

**Table 2.** Per-Unit Spending and Potential Savings Associated With Switching ER Formulations to Generic IR Formulations in Medicaid, 2017

Medication Type, ER Drug Name	ER Formulation				IR Formulation			
	Daily Dose Frequency, Pills/d	Pills Dispensed, No. in Thousands	Mean Spending per Pill, $US	Spending, $ in Millions	Generic Name	Daily Dose Frequency, Pills/d	Mean Spending per Dose Unit, $	Estimated Savings, $US in Millions
**Cardiometabolic**								
Coreg CR	1	296	9.13	2.7	Carvedilol phosphate	2	0.10	2.0
Fluvastatin ER	1	25	5.37	0.1	Fluvastatin sodium	1-2	3.52	0.0
Glipizide ER	1	14 504	0.29	4.3	Glipizide	1-2	0.09	1.6
Glipizide XL	1	9651	0.29	2.8				1.0
Glucotrol XL	1	12	2.18	0.0				0.0
Isosorbide mononitrate ER	1	25 752	0.32	8.1	Isosorbide mononitrate	1-2	0.23	-3.3
Fortamet	1	40	33.96	1.4	Metformin HCl	1-2	0.08	1.0
Glucophage XR	1	84	1.01	0.1				0.1
Glumetza	1	115	78.71	8.6				7.0
Metformin HCl ER	1	122 806	0.58	53.5				44.9
Actoplus Met XR	1	28	15.33	0.4	Pioglitazone HCl plus metformin HCl	1-2	1.97	0.2
Propafenone HCl ER	2	272	5.21	1.4	Propafenone HCl	3	0.32	2.2
**Central Nervous System**								
Dexmethylphenidate HCl ER	1	14 546	5.27	76.6	Dexmethylphenidate HCl	2	0.80	46.3
Focalin XR	1	26 780	11.45	306.6				198.3
Dexedrine	1-2	99	17.30	1.7	Dextroamphetamine sulfate	1-3	1.35	1.1
Dextroamphetamine sulfate ER	1-2	2605	2.89	7.6				0.4
Fluvoxamine maleate ER	1	862	7.17	6.2	Fluvoxamine maleate	2	0.36	4.8
Galantamine ER	1	50	2.33	0.1	Galantamine HBr	2	1.34	0.0
Lamictal XR	1	1430	22.94	31.9	Lamotrigine	2	0.19	24.8
Lamotrigine ER	1	5441	7.50	39.5				33.7
Keppra XR	1	2365	7.61	17.6	Levetiracetam	2	0.23	12.9
Levetiracetam ER	1	9166	0.58	5.2				0.9
Lithium carbonate ER	2	28 091	0.31	8.6	Lithium carbonate	3	0.13	5.7
Lithobid	2	180	6.89	1.2				1.8
Namenda XR	1	836	12.61	10.5	Memantine HCl	1-2	0.64	7.2
Oxtellar XR	1	2538	11.24	29.0	Oxcarbazepine	2	0.36	20.4
Paroxetine CR	1	710	3.84	2.7	Paroxetine HCl	1	0.23	2.2
Paroxetine ER	1	1486	3.80	5.7				4.6
Paxil CR	1	66	6.01	0.4				0.3
Quetiapine Fumarate ER	1	8363	11.26	94.3	Quetiapine fumarate	2	0.37	76.6
Seroquel XR	1	9420	18.16	171.4				125.5
Qudexy XR	1	173	16.27	2.7	Topiramate	1-2	0.18	2.1
Topiramate ER	1	525	11.24	5.7				5.0
Trokendi XR	1	1947	19.11	37.2				28.0
Ambien CR	1	73	13.61	1.0	Zolpidem tartrate	1	0.20	0.7
Zolpidem tartrate ER	1	2824	$1.75	4.9				3.8

Abbreviations: CR, controlled release; ER, extended release; HBr, hydrobromide; HCl, hydrochloride; IR, immediate release; XR, extemded release.

In 2017, the estimated reduction for switching from brand-name ER formulations to generic ER formulations was $299 million. In contrast, switching all ER formulations to generic IR formulations was associated with a reduction of $836 million. Between 2012 and 2017, Medicaid spent $5.9 billion on ER formulations. Switching all patients on ER formulations to generic IR formulations was estimated to be associated with a $5.2 billion spending reduction during the study period ($3.7 billion with rebates adjustment) (Figure 2B). Between Medicare and Medicaid, the combined spending reduction by substituting all ER formulations to generic IR formulations was $2.6 billion in 2017 and $13.7 billion over the study period.

### Partial Interchange Sensitivity Analysis

**Figure 3.**
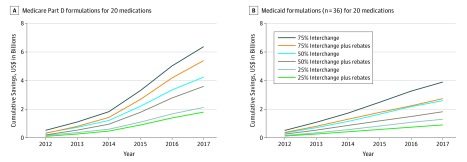
Medicare Part D and Medicaid Cumulative Savings Using Partial Extended-Release to Immediate-Release Interchange With and Without Rebates

In our sensitivity analysis, the cost reduction to Medicare Part D was $6.4 billion ($5.4 billion with rebates) for 75% of drugs, $4.25 billion ($3.6 billion with rebates) for 50% of drugs, and $2.1 billion ($1.8 billion with rebates) for 25% of drugs if switching from an ER formulation to a generic IR formulation occurred (Figure 3A). Similarly, the cumulative cost reduction to Medicaid was $3.9 billion ($2.7 billion with rebates) if a 75% interchange occurred, $2.6 billion ($1.8 billion with rebates) if a 50% interchange occurred, and $1.3 billion ($0.9 billion with rebates) if a 25% interchange occurred (Figure 3B).

### Per-Claim Sensitivity Analysis

#### Medicare Part D

In 2017, the estimated spending reduction for switching from the brand-name ER formulation to the generic ER formulation was $222 million ($183 million with rebates). In contrast, switching all (brand and generic) ER formulations to generic IR formulations in 2017 was associated with a $1.8 billion reduction ($1.6 billion after accounting for brand-name drug rebates) (eTable 1 in the Supplement). Between 2012 and 2017, Medicare Part D spent $12 billion on ER formulations, and switching all patients on ER formulations to generic IR formulations was associated with an $8.9 billion reduction in spending ($8.4 billion after correction for brand-name drug rebates). The absolute spending reduction difference between Medicare per-claim and per-unit analysis was $24 million (1.3%) in 2017 and $386 million (4.5%) between 2012 and 2017.

#### Medicaid

In 2017, the estimated reduction for switching from a brand-name ER formulation to a generic ER formulation was $325 million ($251 million with rebates). In contrast, switching all ER formulations to generic IR formulations was associated with a reduction of $856 million ($682 million with rebates). Between 2012 and 2017, Medicaid spent $6.3 billion on ER formulations. Switching all patients receiving ER formulations to generic IR formulations was estimated to be associated with a $5.5 billion spending reduction during the study period ($4.3 billion with rebate adjustment). The spending reduction difference between Medicaid per-claim and per-unit analysis was $20 million (2.4%) in 2017 and $319 million (6.1%) between 2012 and 2017.

## Discussion

We quantified Medicare Part D and Medicaid spending associated with prescription of ER formulations and found a total potential annual cost savings of up $2.6 billion that could be achieved by switching these drugs to therapeutically comparable generic IR formulations in both Medicare and Medicaid. These savings were demonstrated using only ER formulations saving at most 1 additional daily dose compared with their generic IR formulation counterpart.

Some ER formulations have superior therapeutic effects (eg, metoprolol succinate for heart failure, tolterodine for overactive bladder, and venlafaxine for depression) or fewer adverse effects (eg, niacin for dyslipidemia, nifedipine for patients with heart failure) compared with their generic IR formulation counterparts. Furthermore, ER formulations may be associated with reduced pill burden and improved patient convenience and medication adherence.^5^ However, most of the existing literature on ER vs IR formulations (eTable 2 in the Supplement) has evaluated therapeutic efficacy rather than medication adherence and has frequently demonstrated therapeutic equivalence. In addition, several drugs with both ER and IR formulations available have had no direct ER vs IR comparison studies performed for adherence or therapeutic efficacy (eTable 2 in the Supplement). A meta-analysis of 23 studies^10^ showed no association between administration frequency and medication adherence. Another meta-analysis of 76 studies^11^ showed an adherence benefit of ER formulations only when the administration frequency was reduced by 2 or more daily doses compared with the corresponding IR formulation. To further address this, we only included ER drugs with 1 fewer daily doses than the IR drug counterpart. Taken together, the existing evidence suggests that therapeutic and adherence-related benefits of ER formulations are medication specific and not consistent across all available ER formulations.

Although the present study focused on costs to the payer attributable to the use of ER formulations, the financial strain for patients associated with the higher costs for these drugs remains a significant but underrecognized challenge.^12,13^ Although fewer pills may be more convenient, the added cost burden of ER vs generic IR formulations may be associated with negative utility for patients and perhaps in cost-related nonadherence.^14,15,16^ Studies^14,15,17^ among Medicare patients have shown that 7% to 16% of patients were nonadherent because of excessive medication costs. Cost-related nonadherence has also been associated with more frequent emergency department visits and poorer health outcomes.^17^

However, estimating out-of-pocket costs for Medicare Part D is difficult because plans are heterogenous and provide varying levels of out-of-pocket benefits to their enrollees. The minimum Medicare Part D plan requires patients to be responsible for 100% of costs below the $415 threshold (initial deductible), 25% of costs up to $3820 (initial coverage limit), between 25% and 37% of costs up to $8140 (coverage gap), and 5% of costs beyond $8140 (catastrophic coverage threshold).^18^ Although most plans offer a greater level of coverage for medications in the form of lower copayments or coinsurance, 92% of enrollees did not reach the catastrophic coverage threshold^19^ and could still find significant out-of-pocket savings when switching to the generic IR formulation. The out-of-pocket costs were less significant among Medicaid beneficiaries because Medicaid only requires copayments of $4.00 to $8.00 for beneficiaries below the 150% federal poverty limit. Among beneficiaries who are above the 150% federal poverty threshold, only nonpreferred drugs have significant out-of-pocket costs because these medications require a 20% copayment. It is possible that the only out-of-pocket difference among Medicaid beneficiaries occurs if a nonpreferred ER formulation is prescribed.

Although out-of-pocket savings are beyond the scope of this analysis, we calculated combined Medicare and Medicaid cost savings of $13.7 billion during 6 years, with switching from ER formulations saving at most 1 additional daily dose compared with their generic IR formulations counterpart. However, achieving these savings would likely take a multipronged effort. First, the use of blister packs or prepackaged pill boxes has been shown to improve adherence and may offset the complexity of the added IR pill burden.^20^ Another option is to permit pharmacist-directed therapeutic exchange. Legislation passed in Arkansas, Idaho, and Kentucky allows for pharmacists to substitute a prescribed drug for another in the same therapeutic class.^21^ This option leverages pharmacist expertise and provides substantial opportunity to reduce cost. The option similarly provides patients the ability to make an informed decision because the patient may most readily see variation in out-of-pocket costs at the pharmacy. In addition, legislative action to reduce the price disparity between ER and IR formulation prices may be needed. In our study, the daily price of generic ER formulations was significantly higher than the daily price of generic IR formulations. Reductions in the difference between generic ER formulations and generic IR formulations through additional rebates, drug price negotiations, or additional market competition may assist in reducing the discrepancy between ER and IR medications. Switching from costlier ER formulations to less costly generic IR formulations, at least for medications that have comparable pill burden with ER vs IR formulations, warrants discussion with patients beyond the potential cost savings to Medicare.

### Limitations

This study has limitations. First, the data sets used in this analysis do not include information on the out-of-pocket costs experienced by Medicare or Medicaid beneficiaries. Although the system savings were apparent, it was unclear whether switching to an IR formulation would increase dose burden while failing to provide any individual financial relief. Second, we could not account for patient-level characteristics, such as previous IR drug nonadherence or complexity of medication regimen, which may have contributed to an ER drug prescription. Although underlying patient factors beyond convenience may be associated with the use of some ER prescriptions, our sensitivity analyses with 25% to 75% interchange rates showed a significant cost reduction even if only a small number of patients switched the type of formulation. In addition, we assumed that patients switching from ER to generic IR formulations would take the maximum IR pills per day even though some IR formulations had a range of doses. Thus, our calculations likely underestimated the potential spending reduction because some patients may receive fewer IR drug daily doses.

## Conclusions

This study suggests that there is high spending burden on Medicare and Medicaid associated with the use of ER formulations and that the potential cost savings may be achieved by substituting therapeutically comparable, less-expensive generic IR formulations. Future studies are needed to assess whether this strategy to reduce medication-related expenditures for Medicare and Medicaid is associated with lower patient out-of-pocket costs and thereby positively affects patient care.

## Notes

Supplement. **eTable 1.** Sensitivity Analysis Using Per Claim Analysis. Spending and Potential Savings of Switching Extended-Release Formulations to Generic Immediate-Release Formulations in 2017 in the Medicare Part D **eTable 2.** Literature Review of Extended-release Vs Immediate-release of All Included Study Drugs
