# Supplementary material for: Economic Burden Associated With Extended-Release vs Immediate-Release Drug Formulations Among Medicare Part D and Medicaid Beneficiaries
Source: JAMA Netw Open. 2020 Feb 28;3(2):e200181. doi: 10.1001/jamanetworkopen.2020.0181 (PMC7049080; doi:10.1001/jamanetworkopen.2020.0181)
Supplement: Supplement. — eTable 1. Sensitivity Analysis Using Per Claim Analysis. Spending and Potential Savings of Switching Extended-Release Formulations to Generic Immediate-Release Formulations in 2017 in the Medicare Part D eTable 2. Literature Review of Extended-release Vs Immediate-release of All Included Study Drugs [file jamanetwopen-3-e200181-s001.pdf]

Supplementary Online Content

Sumarsono A, Sumarsono N, Das SR, Vaduganathan M, Agrawal D, Pandey A. Economic burden associated with extended-release vs immediate-release drug formulations among Medicare Part D and Medicaid beneficiaries. *JAMA Netw Open*. 2020;3(2):e200181. doi:10.1001/jamanetworkopen.2020.0181

eTable 1. Sensitivity Analysis Using Per Claim Analysis. Spending and Potential Savings of Switching Extended-Release Formulations to Generic Immediate-Release Formulations in 2017 in the Medicare Part D

eTable 2. Literature Review of Extended-release Versus Immediate-release of all Included Study Drugs

This supplementary material has been provided by the authors to give readers additional information about their work

© 2020 Sumarsono A et al. *JAMA Network Open.*

eTable 1. Sensitivity Analysis Using Per Claim Analysis. Spending and Potential Savings of Switching Extended-Release Formulations to Generic Immediate-Release Formulations in 2017 in the Medicare Part D

Extended Release					Immediate Release			
**Brand/Generic ER Name**	**Daily Dosing Frequency, ER**	**Claims, Thousands**	**Mean Spending per Claim**	**Spending, $ millions**	**Generic Name**	**Daily Dosing Frequency, IR**	**Mean Spending per Claim**	**Estimated Savings, $ millions**
Cardiometabolic Mediations								
Coreg Cr	1	127	$507	$64	Carvedilol Phosphate	2	$7.95	$63.29
Fluvastatin ER	1	21	$310	$7	Fluvastatin Sodium	1 to 2	$194.26	$2.47
Lescol XL	1	5	$523	$3				$1.68
Glipizide ER	1	1,622	$21	$34	Glipizide	1 to 2	$7.46	$21.69
Glipizide XL	1	967	$21	$20				$12.91
Glucotrol XL	1	7	$165	$1				$1.06
Isosorbide Mononitrate ER	1	5,616	$18	$102	Isosorbide Mononitrate	1 to 2	$16.97	$6.36
Glucophage XR	1	12	$150	$2	Metformin HCl	1 to 2	$7.62	$1.68
Glumetza	1	10	$7,619	$74				$73.68
Metformin HCl ER	1	5,551	$58	$320				$277.83
Actoplus Met XR	1	3	$1,057	$3	Pioglitazone HCl/Metformin HCl	1 to 2	$218.23	$2.14
Propafenone HCl ER	2	82	$538	$44	Propafenone HCl	3	$54.24	$39.86
Rythmol SR	2	3	$1,253	$4				$3.92
Central Nervous System Medications								
Dexmethylphenidate HCl ER	1	18	$195	$3	Dexmethylphenidate HCl	2	$51.37	$2.55
Focalin XR	1	2	$490	$1				$1.04
Zenzedi	1 to 2	1	$375	$0.4	Dextroamphetamine Sulfate	1 to 3	$144.31	$0.26
Dextroamphetamine Sulfate ER	1 to 2	49	$225	$11				$4.00
Fluvoxamine Maleate ER	1	20	$382	$8	Fluvoxamine Maleate	2	$30.51	$7.19
Galantamine ER	1	151	$115	$17	Galantamine HBr	2	$98.86	$2.43
Lamictal XR	1	21	$1,900	$40	Lamotrigine	2	$12.23	$40.03
Lamotrigine ER	1	90	$521	$47				$45.74
Keppra XR	1	21	$1,177	$25	Levetiracetam	2	$26.82	$24.31
Levetiracetam ER	1	132	$68	$9				$5.47
Lithobid	2	2	$881	$2	Lithium Carbonate	3	$8.17	$2.18
Lithium Carbonate ER	2	437	$19	$8				$4.75
Namenda XR	1	2463	$362	$891	Memantine HCl	1 to 2	$62.01	$738.49
Oxtellar XR	1	14	$823	$12	Oxcarbazepine	2	$39.77	$11.12
Paroxetine Cr	1	32	$178	$6	Paroxetine HCl	1	$11.71	$5.30
Paroxetine ER	1	88	$192	$17				$15.81

© 2020 Sumarsono A et al. *JAMA Network Open.*

Paxil Cr	1	8	$342	$3				$2.76
Quetiapine Fumarate ER	1	282	$451	$127	Quetiapine Fumarate	2	$17.69	$122.34
Seroquel XR	1	262	$719	$188				$183.54
Qudexy XR	1	2	$848	$2	Topiramate	1 to 2	$13.29	$1.87
Topiramate ER	1	6	$620	$4				$3.78
Trokendi XR	1	24	$977	$24				$23.28
Ambien Cr	1	17	$518	$9	Zolpidem Tartrate	1	$9.26	$8.46
Zolpidem Tartrate ER	1	351	$62	$22				$18.62

© 2020 Sumarsono A et al. *JAMA Network Open.*

eTable 2. Literature Review of Extended-release Versus Immediate-release of All Included Study Drugs

Generic Name	Author, Year	Journal	Number of Patients	Comparison Groups	Patient Population	Primary Endpoints	Adherence Evaluated	Conclusions (> superior to; < inferior to; = equivalent to)
Carvedilol Phosphate	Udelson, 2009	J Card Fail	405	ER vs IR	Heart Failure	Adherence	Yes	CR = IR
Fluvastatin Sodium	Olsson, 2001	Clin Ther	1183	ER vs IR once daily QPM vs IR B.I.D.	Primary hypercholesterolemia	Percentage reduction from baseline in LDL Cholesterol (LDL-C) levels, total cholesterol , HDL cholesterol (HDL-C), Triglycerides, LDL:HDL ratio, apo A 1, and apo B	No	ER = IR B.I.D. > IR QPM
	Isaacsohn, 2003	Clin Ther	173	ER QD dosing vs IR QD dosing	hypercholesterolemia	total cholesterol, LDL-C, triglyceride, apolipoprotein B, HDL-C, and apo A-I levels	No	80mg ER QD > 40mg IR QD
Galantamine	Brodaty, 2005	Dement Geriatr Cogn Disord	971	ER vs IR vs Placebo	Mild-moderate Alzheimer's	Alzheimer's Disease Assessment Scale, Clinician's Interview-Based Impression of Change plus caregiver input	No	ER = IR
Glipizide	Hsieh, 2006	Clin Ther	57	ER vs IR	Type 2 Diabetes	Fasting blood glucose and hemoglobin A1C	No	Intention to treat: ER>IR; fasting blood glucose, ER = IR; hemoglobin

© 2020 Sumarsono A et al. *JAMA Network Open.*

								A1C; Per-protocol analysis: ER = IR in fasting blood glucose and A1C
	Dezii, 2002	South Med J	992	ER Vs IR	T2DM	Adherence	Yes	ER > IR
Levetiracetam	Wu, 2018	Seizure	58	ER vs IR	Uncontrolled Partial Epilepsy	Median partial seizures per week	No	ER = IR
Lithium Carbonate	Durbano, 2002	Clin Ther	39	ER vs IR	Bipolar	Efficacy And tolerability	No	ER with fewer side effects
Metformin	Aggarwal, 2018	Diabetes Obes Metab	539	ER vs IR	T2DM	12 week A1C	No	ER = IR
	Derosa, 2017	Drug Des Devel Ther	253	ER vs IR	T2DM	6 month A1C	No	ER superior to IR
	Schwartz, 2006	Diabetes Care	750	ER vs IR	T2DM	24 week A1C	No	ER = IR
Oxcarbazepine	Bialer,2007	Epilepsy Research	221	CR vs IR	Partial onset epilepsy	Seizure-free subjects	No	ER = IR
Paroxetine HCl	Keene, 2005	Am J Manag Care	604	CR vs IR	Anxiety disorders	Adherence	Yes	ER > IR
Quetiapine Fumarate	Eriksson, 2012	Ther Adv Psychopharmacol	178	Retrospective Observational, XR vs IR	Schizophrenia	Adherence	Yes	XR > IR
	Riedel, 2015	Schizophr Res	65	Crossover XR/IR	Schizophrenia	Cognitive Function and Safety	No	XR = IR
	Riesenberg, 2012	Clin Ther	139	Double Blind Parallel Group	Bipolar	Sedation Intensity and Overall Tolerability	No	XR = IR
	Datto, 2009	Clin Ther	63	Double Blind Crossover	Bipolar	Self-Reported Sedation Intensity by Visual Analog Scale	No	Sedation less intense with XR compared to IR
Dexmethylphenidate	There are no ER vs IR trials							
Dextroamphetamine Sulfate	There are no ER vs IR trials							
Fluvoxamine Maleate	There are no ER vs IR trials							
Isosorbide Mononitrate	There are no ER vs IR trials							
Lamotrigine	There are no ER vs IR trials							
Memantine	There are no ER vs IR trials							

© 2020 Sumarsono A et al. *JAMA Network Open.*

Pioglitazone/Metformin HCl	There are no ER vs IR trials							
Propafenone HCl	There are no ER vs IR trials							
Topiramate	There are no ER vs IR trials							
Zolpidem Tartrate	There are no ER vs IR trials							
Abbreviations: T2DM – Type 2 Diabetes Mellitus; ADHD – Attention Deficit/Hyperactive Disorder								

© 2020 Sumarsono A et al. *JAMA Network Open.*
